# Hypnosis as neurophenomenology

**DOI:** 10.3389/fnhum.2013.00469

**Published:** 2013-08-15

**Authors:** Michael Lifshitz, Emma P. Cusumano, Amir Raz

**Affiliations:** ^1^Integrated Program in Neuroscience, McGill UniversityMontreal, QC, Canada; ^2^Department of Cognitive Science, McGill UniversityMontreal, QC, Canada; ^3^Department of Psychiatry, McGill UniversityMontreal, QC, Canada; ^4^Lady Davis Institute for Medical Research, Jewish General HospitalMontreal, QC, Canada

**Keywords:** hypnosis, suggestion, consciousness, attention, contemplative practice

## Abstract

Hypnosis research binds phenomenology and neuroscience. Here we show how recent evidence probing the impact of hypnosis and suggestion can inform and advance a neurophenomenological approach. In contrast to meditative practices that involve lengthy and intensive training, hypnosis induces profound alterations in subjective experience following just a few words of suggestion. Individuals highly responsive to hypnosis can quickly and effortlessly manifest atypical conscious experiences as well as override deeply entrenched processes. These capacities open new avenues for suspending habitual modes of attention and achieving refined states of meta-awareness. Furthermore, hypnosis research sheds light on the effects of suggestion, expectation, and interpersonal factors beyond the narrow context of hypnotic procedures. Such knowledge may help to further foster phenomenological interviewing methods, improve experiential reports, and elucidate the mechanisms of contemplative practices. Incorporating hypnosis and suggestion into the broader landscape of neurophenomenology, therefore, would likely help bridge subjective experience and third-person approaches to the mind.

## Introduction

Hypnosis and other forms of suggestion hold great promise for advancing the synthesis of phenomenology and cognitive neuroscience. While scientists have access to a plethora of advanced methods for imaging and modeling the brain, first-person procedures for investigating subjective experience lag behind. Anticipating this methodological crisis, in the 1990s Francisco Varela proposed “a quest to marry modern cognitive science and a disciplined approach to human experience” (Varela, 1996). He coined this approach “neurophenomenology”: a mission to establish a method within the cognitive sciences for studying first-person experience alongside third-person accounts of cognition. Neurophenomenology involves a threefold experimental challenge: (1) helping participants generate and sustain specific experiential states, (2) fostering meta-awareness of these states, and (3) collecting accurate first-person descriptions of those states (Lutz and Thompson, 2003). From there, researchers can analyze such qualitative data alongside behavioral and neurobiological data, eventually establishing “strong reciprocal constraints between phenomenological accounts of experience and cognitive-scientific accounts of mental processes” (Lutz and Thompson, 2003, p. 48). Here we propose that cognitive scientists can harness hypnosis and related forms of suggestion to meet the challenges of neurophenomenology. We outline recent advances in hypnosis research to illuminate how this powerful top-down instrument provides a means of (1) generating specific and reliable alterations in consciousness, (2) achieving first-person awareness of the living stream of experience, and (3) elucidating phenomenological interviewing methods to facilitate experiential reporting. Thus, hypnosis offers a largely untapped resource for actualizing the neurophenomenological project.

In recent years, cognitive scientists have increasingly turned to contemplative practices to shed light on both ordinary and atypical cognition. We recently published a special issue synthesizing empirical studies of hypnosis and meditation to present a fresh integrative perspective on contemplative science (Lifshitz and Raz, 2012). In addition to elucidating the empirical correlates of conscious experience, such practices by their very nature call researchers to address the qualitative lived experience of the subject (Thompson, 2006). Some of the earliest proposals for a neurophenomenological method drew heavily on meditative techniques geared at refining attentional skills and improving awareness of the ongoing contents of experience (Varela, 1996). Yet, while altered states of consciousness and contemplative practices have long occupied a central place in neurophenomenology, hypnosis *per se* has received relatively little attention as an exemplar of this approach. A mounting body of scientific evidence has demonstrated that hypnotic suggestions can produce remarkable alterations in subjective experience as well as cognitive and brain function. While historically hypnosis was associated with a special state of consciousness known as “trance,” scientists today disagree concerning the cognitive, behavioral, and neurobiological markers of an ostensible hypnotic state (Oakley and Halligan, 2009, 2010). Regardless of lingering spats concerning the importance of induction rituals and the notion of an “altered state,” however, the collective data clearly demonstrate that hypnotic suggestions can profoundly alter perception, emotion, thought, and behavior, as well as associated brain functions (Kihlstrom, 2008). Among responsive—i.e., highly suggestible—individuals, hypnosis provides a means of rapidly and consistently generating a wide range of uncommon subjective experiences. Hypnosis therefore stands as a promising vehicle for investigating otherwise elusive dimensions of consciousness.

## Reliably inducing specific mind states and experiences

Hypnotic suggestions allow researchers to generate and study atypical experiential states that would otherwise prove unstable, rare, or short-lived. One fruitful approach employs hypnotic suggestions to produce “virtual patients” with transient syndromes nearly identical to genuine clinical psychopathologies in terms of experiential substrates and in some cases also neurobiological correlates (Oakley and Halligan, 2009; Woody and Szechtman, 2011). For example, one study used hypnotic suggestion to produce compelling experiences of mirrored-self misidentification—a clinical condition wherein patients no longer recognize their own reflection in a mirror. Following a suggestion that “the person you see in the mirror will not be you, it will be a stranger,” highly suggestible subjects failed to recognize their own reflection and retained their delusional beliefs in the face of verbal challenges (e.g., “How is it possible that the person in the mirror looks just like you?”) as well as behavioral demands (e.g., being asked to touch their nose while staring in the mirror) (Barnier et al., 2008). We speculate that closer phenomenological investigation of such hypnotically induced distortions of self-perception could reveal nuances concerning the relation between embodied subjectivity and the self viewed as external object (cf. Rochat and Zahavi, 2011). Beyond delusions of mirrored-self recognition, cognitive scientists have employed hypnotic suggestion to generate a wide range of virtual syndromes including obsessive-compulsive disorder (Woody and Szechtman, 2011), synesthesia (Cohen Kadosh et al., 2009), alien-hand syndrome (Blakemore et al., 2003), and visuospatial neglect (Priftis et al., 2011). In addition to the obvious practical advantages of studying virtual rather than genuine clinical patients, hypnotic analogs have the added benefit of allowing researchers to generate subtle nuances in symptomology, as well as design and implement novel delusions and psychopathologies that suit their specific research questions. More broadly, this approach points to the great flexibility afforded by hypnosis to rapidly and consistently modulate deep-rooted structures of experience.

Beyond inducing transient psychopathological symptoms, hypnosis offers insight into spontaneous cognition and the resting brain. Contemplative practices including hypnosis are emerging as valuable tools for investigating the default-mode—a network of brain regions that show increased activity at rest. Default-mode network (DMN) activity correlates with a wide range of internally directed cognitive processes, including mind-wandering, self-oriented thinking, moral reasoning, and episodic memory (Buckner et al., 2008); yet, it is difficult to experimentally manipulate the DMN alongside these processes because the defining feature of the DMN is that it activates spontaneously, in the absence of external task demands. Accordingly, in the past few years, researchers have begun employing contemplative practices in concert with intrinsic connectivity imaging methods to elucidate the psychological correlates of resting-state brain networks such as the DMN (e.g., McGeown et al., 2009; Brewer et al., 2011; Pyka et al., 2011; Hasenkamp et al., 2012; Pagnoni, 2012; Taylor et al., 2013) Following this approach, a recent study reported that hypnotic induction increased subjective ratings of attentional absorption and decreased ratings of mind-wandering. Moreover, these changes were associated with decreased DMN activity and increased activity in prefrontal attention networks (Deeley et al., 2012). Another recent account leveraged a similar neurophenomenological approach to show that subjective ratings of hypnotic depth following an induction were associated with changes in global functional connectivity in the electroencephalography signal. Furthermore, differences in subjective experiential dimensions such as “imagery,” “everyday concerns,” and “vestibular and other bodily experiences” were associated with distinct patterns of connectivity (Cardeña et al., 2013). These studies illustrate how manipulating the experiential state of the subject while collecting subjective reports can enrich and even guide the investigation of brain networks and their psychological correlates.

## Suggesting meta-awareness

Studying subjective experience entails tuning the mind to witness itself. The concept of meta-awareness—i.e., explicit awareness of the contents of one's own ongoing experience—is therefore central to neurophenomenology. The major thinkers associated with the 20th century philosophical movement of Continental Phenomenology struggled continually with the challenge of how to observe and describe experiences whilst living through them subjectively. Synthesizing these early philosophical efforts with meditation techniques derived from contemplative wisdom traditions (i.e., Buddhism and Yoga), recent accounts have begun to develop cognitive models of phenomenological inquiry and practical techniques for developing meta-awareness (Depraz et al., 2000, 2003; Steinbock, 2004). These practices typically involve subduing involuntary conceptualization and verbal overshadowing processes that would otherwise interfere with a more reflexive, embodied awareness of the experiential field. For the scientist advancing a neurophenomenological approach, a crucial challenge is to enable the research participant to foster this kind of open receptive awareness. We propose that hypnosis may provide a valuable tool for helping subjects suspend narrative judgments, loosen deep-seated patterns of conceptual elaboration, and sustain clear and perceptive meta-awareness in an experimental setting.

Meta-awareness seems to require a profound shift in our common modes of attention—calling us to withhold discursive conceptual elaboration and allow experiences to reveal themselves as they are. Cultivating this open receptivity to experience presents a formidable challenge, however, because our patterns of discursive overlay and absorption in mental contents are overlearned and highly automatic. Genuine meta-awareness, therefore, may require powerful first-person tools for overriding habitual preoccupation with particular contents of experience and holding attention open to the ongoing process of experience or the relationship between particular experiences. In this regard, hypnosis may prove useful because it allows highly suggestible individuals to derail processes typically considered ballistic and impervious to willful intervention (Lifshitz et al., 2013). For example, following a hypnotic suggestion to see in black and white, highly responsive individuals perceived brightly colored images in grayscale, with concomitant dampening of low-level brain regions associated with color processing (Kosslyn et al., 2000). Another example involves the classic Stroop paradigm, wherein participants typically demonstrate a lag when asked to report the ink color of incongruent color words (e.g., the word “blue” printed in red) (Stroop, 1935). Based on the robustness of this Stroop interference effect, most cognitive scientists consider processing printed linguistic stimuli inevitable for skilled readers (Macleod, 1991); however, a string of reports from multiple independent laboratories demonstrate that a suggestion to view the stimulus words as meaningless symbols of a foreign language allows participants to override the automaticity of reading and substantially reduce, or in some cases even eliminate, the Stroop interference effect (Raz et al., 2002, 2003, 2006, 2007; Raz and Campbell, 2011; Augustinova and Ferrand, 2012; Parris et al., 2012). Neuroimaging assays have begun to unravel the mechanisms of de-automatization as a function of suggestion (Raz et al., 2005; Casiglia et al., 2010; Terhune et al., 2010), while behavioral accounts have extended these effects to related cognitive paradigms probing automatic visual attention (Iani et al., 2006, 2009) as well as ballistic multimodal perceptual integration (Lifshitz et al., 2013). Appropriate suggestions, therefore, may allow highly suggestible individuals to override automatic conceptual processes that would otherwise interfere with clear phenomenological awareness.

Although hypnosis typically involves suggestions emphasizing specific, and often unusual, behaviors or perceptual experiences (e.g., hallucinations), the defining feature of hypnosis is the flexibility it affords for modulating aspects of consciousness, rather than one particular state of attention that it induces. Indeed, numerous cognitive and neuroimaging reports have documented the power suggestion wields over attention functions and associated brain networks (Egner et al., 2005; Raz et al., 2005; Iani et al., 2006, 2009; Priftis et al., 2011; Raz and Campbell, 2011; Terhune et al., 2011). Such potent subjective alterations could potentially be adapted to derail habitual patterns of conceptual judgment and support non-judgmental meta-awareness of the present moment. Investigators have already begun experimenting with using suggestion-based approaches to foster states of mindful meta-awareness for therapeutic purposes (Lynn et al., 2006, 2010). Yet, efforts to improve meta-awareness via targeted suggestion are still nascent, and the precise wording of such suggestions would require fine-tuning based on the specific goals of the investigator. Classical descriptions of phenomenological awareness as well as traditional meditation instructions would constitute a rich point of departure. Suggestions may be as simple as, for example, noting without judgment the arising and passing of thoughts, emotions, and sensations on a moment-to-moment basis (Lynn et al., 2012).

A subjective sense of effortlessness commonly accompanies hypnotic response, rendering suggestion particularly well suited to promoting meta-awareness. Phenomenologists have often remarked that while one can consciously cultivate a ground ripe for phenomenological insight, the moment of awareness itself entails releasing effortful strategies that would otherwise obscure the phenomena under investigation (Depraz et al., 2000). Acting in accordance with hypnotic suggestions, subjects generally report experiencing their thoughts and actions as effortless and involuntary, as though “the cognitive module that executes the suggestion does so outside of phenomenal awareness” (Kihlstrom, 2008). Dissociative theories of hypnosis characterize hypnosis as a disruption between the cognitive mechanisms responsible for executive control and executive monitoring, respectively (Woody and Sadler, 2012). Thus, working below the level of conscious effort, a suggestion for improved phenomenological awareness may allow practitioners to notice their experience while minimizing interference from the willful act of observation.

One might object that because hypnotic procedures generally involve deep relaxation and mental absorption (Rainville and Price, 2003), phenomenological reports following suggestion would conflate the abnormal processes inherent in hypnotic states with the processes involved in usual waking consciousness. Atypical conscious states, however, need not accompany response to suggestion; although common, relaxation, and fixed attention are unnecessary for propelling responses associated with hypnotic suggestion (Oakley and Halligan, 2010). Indeed, hypnotic phenomena frequently follow even in the absence of an induction ritual or explicit mention of the context of hypnosis (Raz et al., 2006; Mazzoni et al., 2009; McGeown et al., 2012) and responses to suggestions during hypnosis correlate strongly with responses to the same suggestions outside of hypnosis (Kirsch and Braffman, 2001). In addition, potential confounding factors associated with the hypnotic ritual can be avoided by means of posthypnotic suggestion—a condition following termination of the hypnotic experience wherein a subject remains compliant to a suggestion made during hypnosis (Raz and Buhle, 2006). Because posthypnotic suggestion functions during common wakefulness, it may allow participants to view their experience untarnished by abnormalities associated with the hypnotic procedure. Hypnotic and posthypnotic suggestion, therefore, constitute potentially fruitful methods of accomplishing receptive observation of fundamental experiential processes.

## Phenomenological interview as hypnotic encounter

Hypnotic elements already tacitly suffuse established neurophenomenological protocols. As we have seen, hypnosis provides a tool for generating specific states of consciousness and potentially improving first-person awareness of those states. The task of neurophenomenology, however, remains incomplete until the subject conveys a descriptive report from within that place of awareness. Thus, in order to study individuals who lack extensive phenomenological training, researchers require a method of helping subjects uncover and discern aspects of their experience that would otherwise remain invisible. Toward this end, phenomenologists have developed a technique called the “explicitation interview,” which incorporates inter-subjective guidance and non-leading suggestions to promote awareness of processes that typically remain implicit and un-seen within the field of experience (Vermersch, 1990, 2009; Petitmengin, 2006). While these methods are firmly grounded in phenomenological theory and research, cognitive scientists seem hesitant to seize upon them as experimental tools. Bridging such phenomenological methods with scientific models of cognitive and neural function would likely help establish the neurophenomenological approach in mainstream cognitive science. Hypnosis may deliver a piece of this missing link. While phenomenologists do not usually characterize the explicitation interview as a form of hypnosis, the two practices seem to share essential features: like hypnosis, the phenomenological interview reflects a social encounter involving attentional transformations brought on by specific suggestions and carefully cultivated expectations. Thus, we propose that the explicitation interview offers a thought-provoking exemplar of hypnosis-as-neurophenomenology.

The explicitation interview represents a form of “guided retrospective introspection” (Maurel, 2009, p. 59). Interviewees take on the “evocation position”—rediscovering bodily feelings associated with the experience they wish to recall and describe (Vermersch, 2009). Qualitative accounts intimate that this embodied explicitation procedure allows untrained individuals to gain awareness of previously obscured aspects of their experience (Maurel, 2009). Such meta-awareness appears to follow from a series of hypnosis-like interpersonal suggestions. During phenomenological interviews, participants exhibit specific behavioral cues (e.g., spontaneous use of open pronouns as opposed to personal pronouns: see Hendricks, 2009) that may indicate an altered state of awareness similar to what some researchers consider hypnotic absorption (Petitmengin, 2006; Hendricks, 2009; Oakley and Halligan, 2009). Comparable to hypnosis, moreover, the phenomenological interview involves a dynamic exchange between an authoritative guide and a willing participant who share explicit goals and expectations concerning the desired results. Numerous studies drawing on hypnosis illuminate how social factors and expectation interact to shape the outcomes of suggestion (Gandhi and Oakley, 2005; Lifshitz et al., 2012). For example, simply labeling a procedure as “hypnosis” strengthens the effects of certain suggestions (Gandhi and Oakley, 2005). Analogous situational cues and attitudes likely mold participant reports during phenomenological interviews. Phenomenologists not only appreciate such socio-cognitive factors but even openly acknowledge suggestion-based research, especially the work of renowned hypnosis pioneer Milton Erickson, as an important influence in developing the explicitation technique (Vermersch, 1990). Further engaging with the extensive and ever-growing hypnosis literature may help neurophenomenologists capitalize on the implicit expectations and suggestive elements inherent in the interview context.

The relationship between memory and awareness is central to phenomenological interviewing. Phenomenologists, however, scarcely address the extensive data illustrating how hypnosis and other forms of suggestion can either improve or bias recall of past experiences. Two important events in the late twentieth century marred popular conceptions of hypnosis in relation to memory: the massive rise in dissociative identity disorder diagnoses (Lynn et al., 2008) and the increasing interest in legal issues surrounding the use of hypnotically enhanced memories in court (Orne, 1979; Timm, 1981; Belli and Loftus, 1996). While these historical misapplications have tainted the image of hypnosis as a useful tool for evoking lost memories, the collective evidence shows that judicious use of hypnotic techniques can actually improve recall and decrease memory inaccuracy. Expectancies and demand characteristics appear to play a primary role in determining whether hypnosis or other suggestion-based procedures will either improve or bias memory (Lynn et al., 2012). A series of recent experiments demonstrates that instructing participants to expect that hypnosis leads to accurate memory reporting increases resilience to misleading information and decreases the likelihood of false recall (Wagstaff et al., 2011). Participants may respond to such expectancies by more closely monitoring the process of recall and raising their threshold regarding which memories feel true enough to report (Koriat and Goldsmith, 1996). By emphasizing non-leading questions and open prompts, explicitation interviewers already appear conscientious of the relationships among suggestion, expectation, and memory; conducting experiments adapted from false memory studies (e.g., Belli and Loftus, 1996), moreover, may help to empirically confirm the validity of elicited reports. Deliberately crafting suggestions that associate the explicitation procedure with correct recall may serve to safeguard against the menace of confabulation and help foster more detailed and accurate experiential reports.

## Conclusion

An overlooked boon to consciousness researchers, hypnosis is unmatched in its capacity to rapidly evoke profound alterations in experience. Hypnotic suggestion provides an efficient and precise tool for meeting three core challenges of neurophenomenology: (1) reliably generating altered states of awareness, (2) fostering meta-awareness, and (3) encouraging accurate and detailed experiential reports. Moreover, hypnosis-like dynamics appear to implicitly permeate current phenomenological practices. As such, first and second-person approaches stand to benefit from openly engaging with the science of suggestion; on the flip side, such subjective methodologies would likely advance the science of hypnosis. Whereas hypnosis research is presently booming with experimental protocols for studying the influence of suggestion on brain and behavior, researchers seldom capitalize on phenomenological methods including the explicitation interview. Such phenomenological methods may serve to elucidate topical issues in hypnosis science. For example, recent efforts have begun to harness first-person reports alongside neuroimaging data to unravel spontaneous cognitive and experiential fluctuations associated with hypnotic induction (Cardeña et al., 2013). These efforts reflect the spirit of mutuality that the neurophenomenological community so faithfully upholds. In line with this reciprocal attitude, braiding together hypnosis science and subjective methodologies promises to enrich our empirical understanding of both while tilling the soil for future explorations of consciousness and cognition.

### Conflict of interest statement

The authors declare that the research was conducted in the absence of any commercial or financial relationships that could be construed as a potential conflict of interest.
